# DCSwinLSTM for spatiotemporal meteorological drought forecasting

**DOI:** 10.1016/j.isci.2026.115902

**Published:** 2026-04-25

**Authors:** Haoxiang Peng, Chengrong Wu, Yuhao Du, Hui Liu

**Affiliations:** 1 School of Computer Science and Mathematics, Central South University of Forestry and Technology, Changsha 410004, China; 2 Department of Computer Science, University of Manchester, Manchester M13 9PL, UK; 3 College of Atmospheric Sciences, Lanzhou University, Lanzhou 730000, China

**Keywords:** Earth sciences, Machine learning, Water resources engineering

## Abstract

Meteorological drought prediction is critical for early warning and climate-risk management; yet, modeling regional drought evolution across multi-timescale remains challenging. Here, we propose DCSwinLSTM, a feature-fusion framework that combines deformable convolution for boundary-sensitive spatial encoding, a Swin transformer for hierarchical multi-scale feature learning, and an LSTM recurrent update for temporal dependency modeling. Using a global gridded dataset (1959–2022) constructed from the standardized precipitation index (SPI) and standardized precipitation evapotranspiration index (SPEI) at 3- and 6-month scales, DCSwinLSTM consistently outperforms mainstream spatiotemporal prediction baselines. On the held-out SPI-3 test set, it achieves mean square error (MSE) 0.4269, mean absolute error (MAE) 0.5050, root mean square error (RMSE) 0.6534, R^2^ 0.5875, and PSNR 20.6669; on SPI-6, it attains MSE 0.2800, MAE 0.4003, RMSE 0.5292, R^2^ 0.7265, and PSNR 22.0230. These results support reliable multi-timescale drought forecasting for risk management and water-resource planning, even in data-scarce regions.

## Highlights

• DCSwinLSTM combines DConv, Swin transformer, and recurrent updates
• Boundary-aware fusion improves drought-patch continuity and accuracy
• DCSwinLSTM-m yields higher accuracy with greater computational cost
• The model shows strong performance on SPI/SPEI and precipitation tasks

## Graphical abstract

**Figure:**
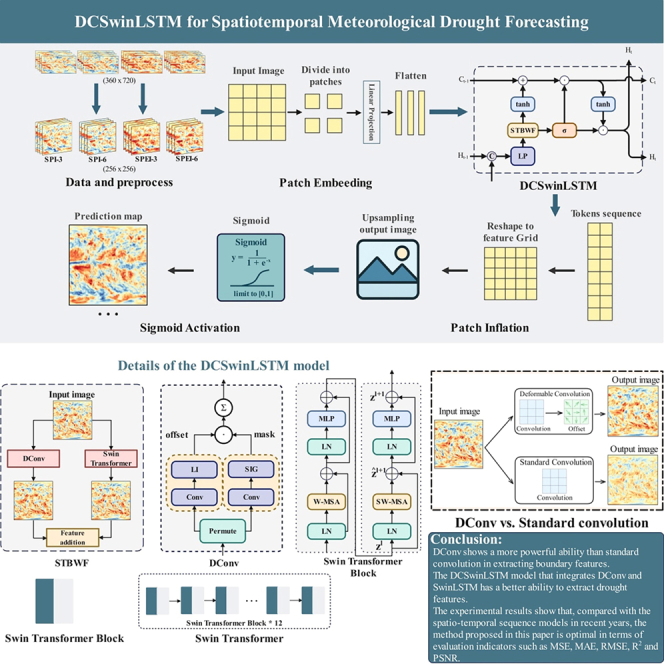

## Introduction

Meteorological drought is a complex hydroclimatic phenomenon that can be intensified under anthropogenic warming through increased atmospheric evaporative demand interacting with precipitation deficits,^1^ Recent global analyses further indicate that the terrestrial area affected by persistent multiyear droughts has increased markedly.^2^ Extreme droughts can have severe impacts on vegetation degradation^3^ and pose a serious threat to food security,^4^ thereby exerting a significant impact on human society. There are numerous factors contributing to meteorological droughts. In addition to meteorological variables such as total precipitation, temperature, wind speed, evapotranspiration, and soil moisture,^5^^,^^6^ geographic factors—including differences in topography, land characteristics, and land use-also play a significant role.^7^ The complex spatiotemporal variations of these factors result in diverse patterns of drought occurrence and distribution across different times and regions.

Currently, research on drought prediction can be broadly categorized into model-driven methods (MDMs), data-driven methods (DDMs), and hybrid approaches that combine elements of both.^8^ MDMs extract meteorological drought signals from dynamical forecasting systems by predicting precipitation and temperature fields and subsequently deriving drought indices. They provide physically consistent evolution and stronger interpretability, but their skill can be limited by structural model errors, imperfect initial conditions, parameterization uncertainty, and systematic biases, which often necessitate statistical post-processing or bias correction.^9^ DDMs, in contrast, treat drought prediction as a statistical learning problem, ranging from classical time-series models such as ARIMA and SARIMA to modern machine learning and deep learning approaches.^10^^,^^11^ These methods can learn nonlinear relationships directly from historical drought-index sequences and related predictors; yet, they may be sensitive to data quality and sample size, vulnerable to overfitting or performance degradation under climate non-stationarity, and typically offer weaker physical interpretability^12^^,^^13^ Hybrid approaches seek to combine the complementary strengths of MDMs and DDMs by calibrating dynamical-model signals with statistical or machine-learning corrections and embedding physically informed constraints into data-driven learning, thereby improving forecast robustness and regional skill for meteorological drought applications.^14^

Within data-driven methods, deep learning has become a major line of research for meteorological drought forecasting because it can learn nonlinear dependencies from long drought-index sequences and multi-source predictors. Early deep-learning studies mainly adopted recurrent neural networks, where LSTM-based models improved temporal dependency modeling through gated memory and were widely used for drought-index time-series prediction.^15^^,^^16^ To further enhance skill and robustness, hybrid designs that couple LSTM with conventional statistical components have also been explored for drought forecasting, aiming to stabilize predictions and better represent evolving drought dynamics.^17^^,^^18^

As drought evolution is inherently spatiotemporal, later advances extended purely temporal learning to spatiotemporal sequence prediction by explicitly modeling spatial structure together with temporal recurrence. ConvLSTM introduced convolutional state transitions to capture local spatial dependence while maintaining recurrent temporal evolution, and it has since served as a foundational backbone for geophysical sequence modeling.^19^ Building on this backbone, memory-augmented recurrent architectures such as PredRNN and MIM were proposed to better represent non-stationary spatiotemporal dynamics and long-range temporal evolution, and these designs have inspired subsequent drought-oriented spatiotemporal predictors.^20^^,^^21^^,^^22^ More recently, efficient CNN-based predictive learners have demonstrated that carefully designed convolutional pipelines can provide competitive spatiotemporal forecasting performance with improved computational efficiency,^23^ while wavelet-enhanced predictive learning further bridges time-frequency variations for complex geophysical sequences.^24^

Despite these advances, convolution-centric recurrence remains constrained by limited receptive fields and difficulty in capturing long-range spatial interactions, which motivates attention-based modeling. Transformer-style architectures, relying on self-attention and parallel sequence processing, have been introduced to improve long-range dependency modeling and have shown promising performance in drought-related prediction tasks.^25^ In particular, Swin transformer provides a hierarchical representation through shifted-window attention, enabling multi-scale spatial modeling while controlling computational cost.^26^ SwinLSTM integrates such hierarchical attention with recurrent temporal modeling, offering a practical framework to jointly learn hierarchical spatial representations and temporal evolution for spatiotemporal forecasting.^27^

Recent studies have shown that meteorological droughts often exhibit distinct and spatially coherent boundary characteristics at the regional scale, shaped by climatic zones, topography, and local circulation patterns.^28^^,^^29^ These boundary-dominated regional patterns can be physically linked to persistent large-scale circulation anomalies (e.g., blocking highs and stationary-wave patterns) that suppress precipitation over broad areas, teleconnection-driven precipitation anomalies, and local geographic constraints such as orography and land-sea contrast that anchor precipitation gradients in space.^30^^,^^31^^,^^32^ Therefore, preserving region-specific boundary continuity is important because small boundary-location errors can translate into large biases in the predicted drought-affected area and the spatial distribution of impacts, especially for regional early-warning applications.^33^^,^^34^ However, most existing prediction models—ranging from statistical and physical approaches to deep learning architectures—still struggle to generalize across diverse regions and to reliably capture these boundary-driven regional trends. This gap highlights a key scientific question: how to represent and forecast spatiotemporal and nonlinear drought evolution while preserving region-specific boundary continuity, so as to reduce uncertainties in regional trend prediction. To address this issue, we replace the equally weighted sampling of standard convolutions with deformable convolution, which learns adaptive offsets to shift each kernel’s sampling points toward drought boundaries and spatially coherent regional patches. Guided by these learned offsets and supported by interpolation, DConv expands and bends its receptive field to follow irregular boundary shapes, assigns greater emphasis to structurally important regions, and preserves boundary continuity—thereby enabling the model to better capture the spatially coherent drought signatures that conventional convolutions tend to dilute. Furthermore, to improve the capture of multi-scale spatiotemporal features of drought, we integrate the powerful global modeling ability of the SwinLSTM model and construct a feature fusion prediction framework to enhance the accuracy and reliability of meteorological drought event prediction.

In this study, SPI and SPEI at different accumulation windows are treated as separate forecasting tasks; for each index and timescale, the model uses four preceding monthly maps to predict the next-month map at the same timescale, i.e., a 1-month lead time. The model is trained and evaluated using 3-month and 6-month SPI and SPEI indices derived from the ERA5 dataset. The results of the selected evaluation metrics indicate that our model achieves significant improvements over existing benchmark spatiotemporal prediction models for meteorological drought prediction at various time scales.

## Results

### Feature fusion with DConv and Swin transformer improves drought-map prediction accuracy

**Figure 1:**
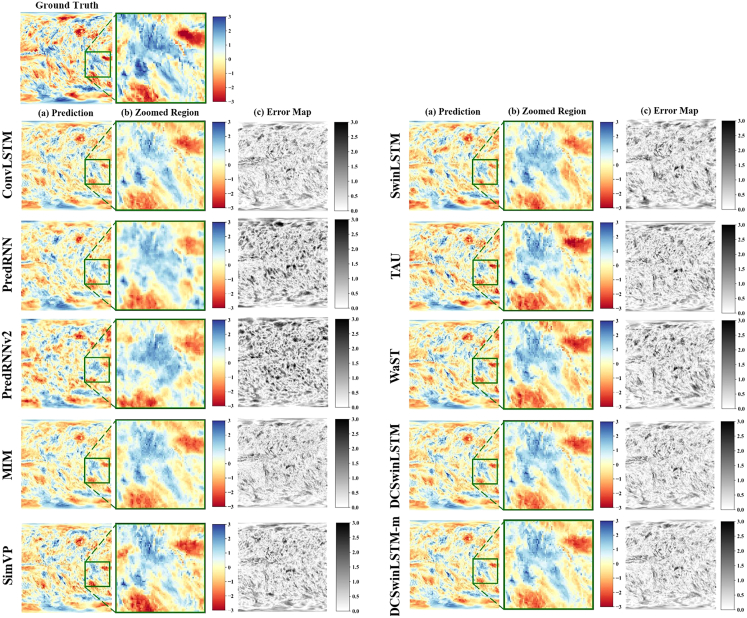
Qualitative comparison of SPI-3 predictions for the target date 2010-06-01 Rows show different models. Columns show (a) model prediction, (b) zoomed-in region (green box) for boundary comparison, and (c) signed error maps.

**Figure 2:**
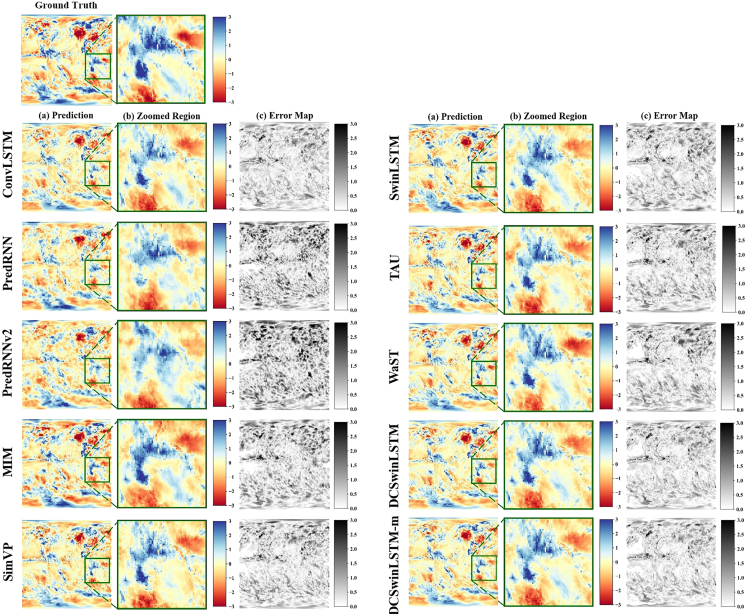
Qualitative comparison of SPEI-3 predictions for the target date 2010-06-01 Rows show different models. Columns show (a) model prediction, (b) zoomed-in region (green box) for boundary comparison, and (c) signed error maps.

In order to compare the performance of different models in drought prediction, we selected ConvLSTM, PredRNN, MIM, PredRNN-V2, SimVP, SwinLSTM, TAU, and WaST as comparison models. Figures 1 and 2 show the three types of images are presented for each experiment for the target date 2010-06-01: the ground truth image, the prediction image, and the error image.The ground truth image is obtained by computing SPI or SPEI index values from the ERA5 dataset after preprocessing (including padding, interpolation and normalization), and then rasterizing them onto a 2-D grid. The prediction image shows the model-predicted SPI/SPEI values rendered on the same 2-D grid. The error image is calculated as the pixel-wise signed difference $E=\hat{y}-y$ (prediction minus ground truth). In these error maps, pixels closer to white or black indicate larger deviations, while pixels closer to neutral tone indicate smaller or near-zero errors. Positive (negative) values indicate overestimation (underestimation), and the colorbar provides the error magnitude and sign. These visualizations highlight the performance differences between the comparison models and the proposed DCSwinLSTM in meteorological drought prediction. Through ground truth and prediction visualization results, it is found that the overall block structure of the predicted area of PredRNN, PredRNN-V2, SimVP, TAU, and WaST models is significantly different from the real value (see the enlarged image in Figures 1 and 2), reflecting their limitations in extracting drought location features, resulting in relatively insufficient prediction accuracy. The boundary of the drought area of SwinLSTM is incomplete, and a small part of the drought area that is supposed to have disappeared. On the contrary, ConvLSTM and MIM are the opposite. A small part of the non-drought area in the drought area is mistakenly predicted as drought. In contrast, the hybrid model proposed in this study that integrates multi-scale attention mechanisms uses deformable convolutions to significantly improve the ability to extract block style features in arid areas while maintaining prediction accuracy. In addition, in the results of the SPEI-3 index (see Figure 2), the fusion model can better capture the block characteristics of the drought area than the results of the SPI-3 index (see Figure 1), thereby improving the accuracy of the prediction.

**Figure 3:**
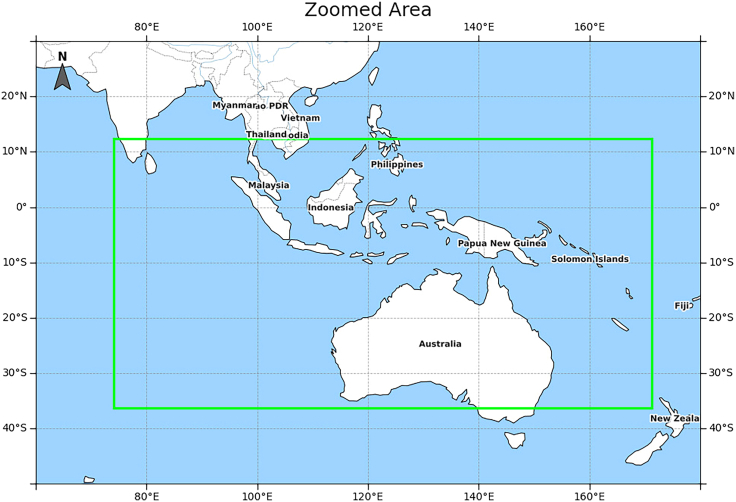
Geographic context of the enlarged region used in the qualitative comparisons The green rectangle outlines the Indo-Pacific domain analyzed in Figures 1 and 2.

To further ground the qualitative comparison in geographic context, we analyze the enlarged region shown in Figures 1 and 2, which is marked in Figure 3 as the selected Indo-Pacific domain spanning the tropical to subtropical belt. This region features strong land-sea contrasts and organized moisture-transport and circulation variability. As a result, drought conditions often appear as coherent patch-like structures with relatively sharp dry-wet transition zones rather than scattered isolated pixels. In this setting, the integrity of drought-patch boundaries is physically meaningful, because boundary displacement corresponds to shifts of the dry-wet transition related to changes in moisture convergence and large-scale circulation. The zoomed comparisons in Figures 1 and 2 show that several baselines tend to fragment or misplace these patch boundaries, while DCSwinLSTM better preserves spatial continuity and the shape of drought patches in this region. This qualitative observation is further examined by our boundary-focused evaluation.

Table 1 to Table 4 respectively give the comparison of evaluation index values of drought prediction tasks under four different drought indices and time scales: SPI-3, SPI-6, SPEI-3, and SPEI-6. Red indicates the best effect, while blue indicates the second-best effect. All metrics are computed after de-normalizing predictions and ground truth back to the original SPI/SPEI units.

**Table 1:** Quantitative comparison of DCSwinLSTM and other methods on SPI-3

Method	MSE↓	MAE↓	RMSE↓	R^2^↑	PSNR↑
ConvLSTM(NeurIPS’2015)	0.4348	0.5112	0.6594	0.5799	20.5873
PredRNN(NeurIPS’2017)	0.7804	0.6942	0.8834	0.2460	18.0471
MIM(CVPR’2019)	0.4676	0.5326	0.6838	0.5470	20.2714
PredRNN-V2 (TPAMI’2022)	0.8506	0.7274	0.9223	0.1782	17.6729
SimVP(CVPR’2022)	0.4941	0.5451	0.7029	0.5227	20.0325
SwinLSTM(ICCV’2023)	0.5119	0.5561	0.7154	0.5055	19.8788
TAU(CVPR’2023)	0.5093	0.5539	0.7136	0.5080	19.9010
WaST (AAAI’2024)	0.5002	0.5483	0.7072	0.5168	19.9793
**DCSwinLSTM(OURS)**	0.4269	0.5050	0.6534	0.5875	20.6669
**DCSwinLSTM-m(OURS)**	0.4196	0.5008	0.6478	0.5946	20.7422

From the results of the four drought prediction tasks, our proposed framework consistently achieves the best or tied-best performance across the reported metrics (Tables 1, 2, 3, and 4). DCSwinLSTM and the variant DCSwinLSTM-m, obtain the top results on SPI/SPEI at 3- and 6-month scales, demonstrating the robustness of the proposed fusion design for multi-scale meteorological drought forecasting. Compared with the baseline model, they yield lower prediction errors (mean-square error [MSE]/mean absolute error [MAE]/root mean square error [RMSE]) and improved fitting and perceptual quality (R^2^/PSNR). In particular, DCSwinLSTM-m delivers further accuracy gains under the same setting, at the cost of a larger parameter budget, providing a higher-performance option when computational resources allow.

**Table 2:** Quantitative comparison of DCSwinLSTM and other methods on SPI-6

Method	MSE↓	MAE↓	RMSE↓	R^2^↑	PSNR↑
ConvLSTM (NeurIPS’2015)	0.2845	0.4039	0.5334	0.7221	21.9546
PredRNN (NeurIPS’2017)	0.5955	0.6058	0.7717	0.4183	18.7459
MIM (CVPR’2019)	0.3191	0.4342	0.5649	0.6874	21.4556
PredRNN-V2 (TPAMI’2022)	0.7142	0.6652	0.8451	0.3023	17.9564
SimVP (CVPR’2022)	0.3465	0.4514	0.5886	0.6616	21.0984
SwinLSTM (ICCV’2023)	0.3156	0.4266	0.5618	0.6917	21.5033
TAU (CVPR’2023)	0.3496	0.4538	0.5913	0.6585	21.0590
WaST (AAAI’2024)	0.3481	0.4506	0.5900	0.6600	21.0779
**DCSwinLSTM (OURS)**	0.2800	0.4003	0.5292	0.7265	22.0230
**DCSwinLSTM-m (OURS)**	0.2775	0.3991	0.5267	0.7290	22.0630

**Table 3:** Quantitative comparison of DCSwinLSTM and other methods on SPEI-3

Method	MSE↓	MAE↓	RMSE↓	R^2^↑	PSNR↑
ConvLSTM (NeurIPS’2015)	0.3948	0.4745	0.6284	0.6350	27.0916
PredRNN (NeurIPS’2017)	0.7397	0.6535	0.8600	0.3162	24.3653
MIM (CVPR’2019)	0.6124	0.5935	0.7826	0.4311	25.1852
PredRNN-V2 (TPAMI’2022)	0.8537	0.7056	0.9240	0.2108	23.7427
SimVP (CVPR’2022)	0.4445	0.4984	0.6667	0.5891	26.5770
SwinLSTM (ICCV’2023)	0.4715	0.5136	0.6866	0.5641	26.3211
TAU (CVPR’2023)	0.4459	0.5000	0.6678	0.5878	26.5633
WaST (AAAI’2024)	0.4463	0.4983	0.6680	0.5874	26.5597
**DCSwinLSTM (OURS)**	0.3932	0.4682	0.6271	0.6365	27.1093
**DCSwinLSTM-m (OURS)**	0.3815	0.4617	0.6176	0.6474	27.2412

**Table 4:** Quantitative comparison of DCSwinLSTM and other methods on SPEI-6

Method	MSE↓	MAE↓	RMSE↓	R^2^↑	PSNR↑
ConvLSTM (NeurIPS’2015)	0.2502	0.3805	0.5002	0.7663	26.2976
PredRNN (NeurIPS’2017)	0.5866	0.5931	0.7659	0.4521	22.5976
MIM (CVPR’2019)	0.2826	0.4067	0.5316	0.7349	25.7694
PredRNN-V2 (TPAMI’2022)	0.7396	0.6687	0.8600	0.3092	21.5911
SimVP (CVPR’2022)	0.3041	0.4210	0.5514	0.7160	25.4511
SwinLSTM (ICCV’2023)	0.2437	0.3747	0.4937	0.7723	26.4114
TAU (CVPR’2023)	0.3095	0.4249	0.5563	0.7109	25.3748
WaST (AAAI’2024)	0.3077	0.4233	0.5547	0.7126	25.3999
**DCSwinLSTM (OURS)**	0.2449	0.3744	0.4948	0.7731	26.3918
**DCSwinLSTM-m (OURS)**	0.2429	0.3738	0.4928	0.7731	26.4266

**Figure 4:**
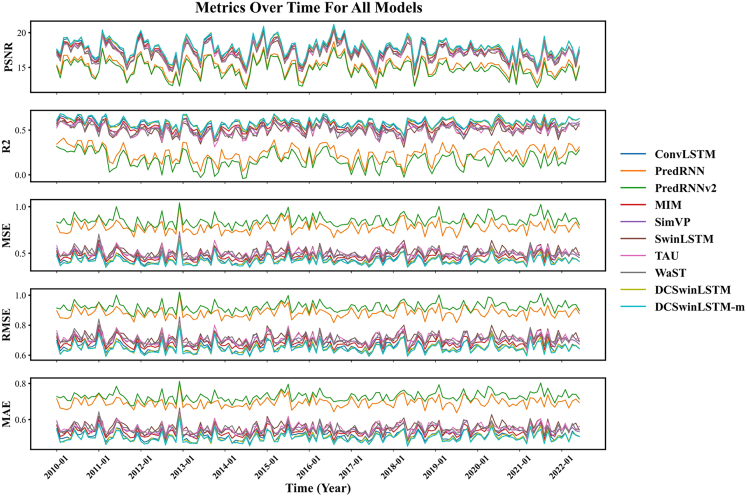
Comparison chart of different model evaluation indicators Using SPI-3 data from January 2010 to June 2022 for prediction and calculating MSE, RMSE, MAE, R2, PSNR to draw line graphs.

**Figure 5:**
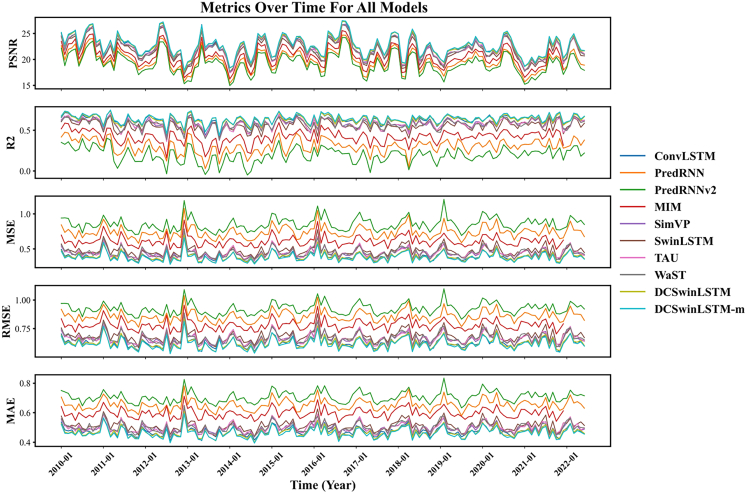
Comparison chart of different model evaluation indicators Using SPEI-3 data from January 2010 to June 2022 for prediction and calculating MSE, RMSE, MAE, R2, PSNR to draw line graphs.

Different models are used to predict the SPI-3 and SPEI-3 indicators from January 2010 to May 2022. The MSE, MAE, RMSE, R^2^, and PSNR of the monthly prediction results are calculated and visualized by comparing the true values and the predicted values. The results are shown in Figures 4 and 5. The graphs show that the model proposed in this paper is ahead of other baseline models in all indicators.

### DCSwinLSTM generalizes from drought-index maps to precipitation-field forecasting

Precipitation anomalies are one of the primary physical drivers of meteorological drought onset and evolution, and they directly underpin precipitation-based drought indices such as SPI. Even for SPEI, which additionally involves evaporative demand, the spatiotemporal organization of rainfall strongly shapes where drought patches emerge, how they expand, and how long they persist. Therefore, validating a model on precipitation-field forecasting provides an informative complementary check for drought studies: it helps assess whether the model learns transferable spatiotemporal structures that are relevant to drought dynamics-such as coherent patch formation, sharp transition bands, and temporally consistent spatial evolution.

**Figure 6:**
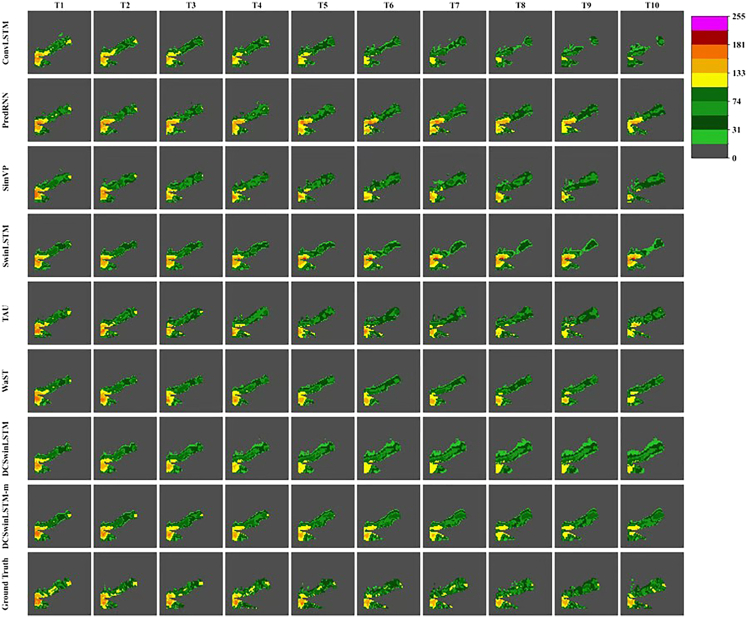
CIKM2017 precipitation forecasting qualitative results for a representative test case Each column denotes a forecast time step (T1–T10), and each row shows the corresponding output from a given method; the bottom row provides the ground-truth sequence. The color bar indicates precipitation intensity, and the same visualization range is applied to all methods for fair comparison.

Motivated by this drought-precipitation linkage, we further evaluate our approach on the CIKM2017 precipitation benchmark to examine generalization beyond SPI/SPEI map forecasting. We follow the standard benchmark protocol reported by Du.^35^ For a fair comparison, the input format and experimental settings (data preprocessing, lead time, and training configuration) also follow Du et al. We report MSE, MAE, and CSI at multiple thresholds (CSI30/CSI40/CSI50). In addition to the quantitative scores, Figure 6 provides representative qualitative comparisons on the CIKM2017 benchmark, showing forecasts from each method across lead times (T1–T10) together with the ground-truth sequence under a consistent visualization range. As summarized in Table 5, DCSwinLSTM achieves the strongest overall performance among the compared methods: it attains the lowest MSE and consistently strong CSI scores, indicating improved reconstruction of precipitation-event patterns and their spatial extent. We also report the multi-gate variant DCSwinLSTM-m in the same setting; it is competitive in MAE but does not surpass the single-gate version on the full set of metrics, suggesting a trade-off between slightly improved magnitude accuracy and overall error. Overall, these precipitation results provide additional evidence that the proposed fusion design captures general spatiotemporal structures that are closely related to drought-relevant dynamics.

**Table 5:** Quantitative results on CIKM2017

Method	MSE↓	MAE↓	CSI30↑	CSI40↑	CSI50↑
ConvLSTM (NeurIPS’2015)	28.8162	161.242	0.7757	0.6590	0.5367
PredRNN (NeurIPS’2017)	28.8126	159.404	0.7803	0.6690	0.5471
MIM (CVPR’2019)	27.2072	154.269	0.7828	0.6725	0.5587
PredRNN-V2 (TPAMI’2022)	28.0085	161.508	0.7857	0.6640	0.5362
SimVP (CVPR’2022)	31.3121	166.261	0.7731	0.6594	0.5416
SwinLSTM (ICCV’2023)	27.9612	158.899	0.7826	0.6652	0.5532
TAU (CVPR’2023)	30.5827	161.948	0.7779	0.6601	0.5370
WaST (AAAI’2024)	30.3074	165.804	0.7773	0.6534	0.5174
**DCSwinLSTM (OURS)**	24.7307	161.091	0.7967	0.6826	0.5604
**DCSwinLSTM-m (OURS)**	27.1285	155.959	0.7909	0.6727	0.5435

### Ablation shows complementary benefits of DConv and Swin transformer for SPI forecasting

To verify the effectiveness of using Dconv and Swin transformer in model improvement, this paper compares the basic SwinLSTM model (only using Swin transformer), the improved single gate DCSwinLSTM (using DConv and Swin transformer), and the standard multi-gate LSTM update variant DCSwinLSTM-m. Similarly, to evaluate the effectiveness of deformable convolutions in extracting boundary features, we replaced standard convolution in ConvLSTM with DConv, yielding the DConvLSTM, In addition, we include a naive FC + LSTM baseline, which flattens each 2D map and applies a fully connected projection before an LSTM, serving as a temporal-only reference without explicit spatial modeling. We limited the ablation experiments to SPI rather than SPEI. On the one hand, SPI relies solely on precipitation data, avoiding the additional noise caused by evapotranspiration estimates in SPEI and more sensitively exposing differences in model structure. On the other hand, early experiments have verified that the performance trends of the two indicators are almost consistent across different models. We used SPI-3 as it provides representative mid-term drought signals for model evaluation while reducing computational load, thereby allowing a focused and efficient assessment of each component’s contribution. The final results are shown in Table 6.

**Table 6:** Ablation experiment on SPI-3

Method	MSE↓	MAE↓	RMSE↓	R^2^↑	PSNR↑
**FC-LSTM**	0.9736	0.7845	0.9867	0.0594	17.0924
**ConvLSTM**	0.4348	0.5112	0.6594	0.5799	20.5873
**DConvLSTM**	0.4301	0.5078	0.6576	0.5841	20.6243
**SwinLSTM**	0.5119	0.5561	0.7154	0.5055	19.8788
**DCSwinLSTM**	0.4269	0.5050	0.6534	0.5875	20.6669
**DCSwinLSTM-m**	0.4196	0.5008	0.6478	0.5946	20.7422

The experimental results show that DCSwinLSTM outperforms the baseline model SwinLSTM, achieving lower errors and better fit, with MAE and RMSE reduced by 9.19% and 8.67%, and R^2^ and PSNR improved by 16.20% and 3.96%, respectively. These results indicate that, in the drought prediction task, the introduction of the DConv module significantly enhances the model performance. Moreover, the naive FC-LSTM baseline performs substantially worse, highlighting that temporal recurrence alone is insufficient once the 2D drought maps are flattened and spatial structure is weakened. In contrast DConvLSTM, which replaces the standard convolution in ConvLSTM with DConv, brings only marginal changes relative to DCSwinLSTM and performance is very close to it overall, showing a small increase in MAE/RMSE (0.74%/0.38%) and a minor drop in R^2^/PSNR (0.35%/0.16%). This further shows that the DConv module needs to work together with the Swin transformer module to fully exploit its advantages, and it is difficult to obtain the best drought prediction effect by using any one module alone, replacing the simplified single-gate update with a standard multi-gate LSTM yields an improvement, suggesting that the full gating mechanism can better capture fine-grained temporal dynamics. For applications that prioritize maximum accuracy, the DCSwinLSTM-m can be adopted; for settings with limited computational resources or efficiency constraints, the single-gate version provides a practical alternative while maintaining competitive performance within our overall architecture.

In addition, commonly used feature fusion methods include add and concat, weighted sum, hadamard product, gating mechanism, etc. Through experiments, it is found that the experimental results of add and concat methods are similar, and the effects of other methods are slightly weaker. However, since the add method has smaller parameter requirements than concat, the feature fusion method chooses additive fusion.

### Visualization shows deformable convolution focuses on drought edges and boundaries

**Figure 7:**
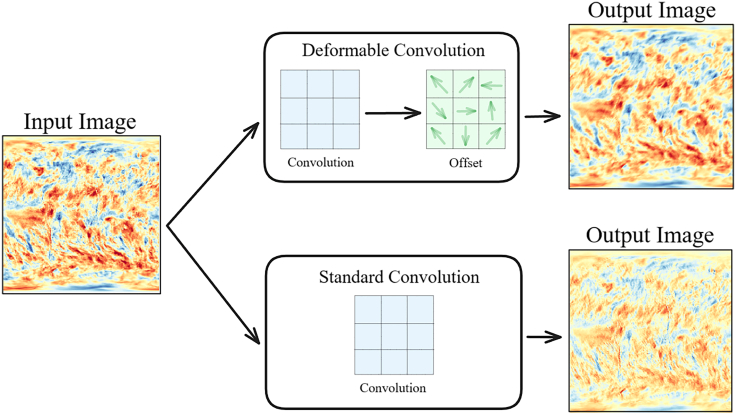
Convolution feature comparison chart The input drought data are extracted through deformable convolution and standard convolution respectively to obtain different feature maps.

After evaluating the overall performance of the model, in order to further analyze and verify the ability of DCSwinLSTM to extract spatial features of arid areas, this study uses drought data as input to visualize the feature extraction effect of DConv. From the input map of Figure 7, it can be found that the arid area shows block characteristics in spatial distribution. This feature shows that the drought phenomenon is not dispersed, but exists in a relatively concentrated area, which may be affected by factors such as climate patterns, topography and land use changes. A careful comparison of the feature maps of DConv and standard convolution shows that the feature maps of standard convolution in block-distributed arid areas show intermittent arid areas, while DConv is more continuous in feature extraction of arid areas. Its DConv enables it to more accurately model the morphology and distribution characteristics of arid areas, and can more specifically extract features for block-distributed arid areas, thereby improving the integrity of feature expression. Moreover, by comparing the color difference between standard convolution and DConv in Figure 7, it can be seen that the color similarity of standard convolution after feature extraction is not as good as that of DConv. In other words, DConv is better than standard convolution in feature extraction data accuracy, which further enhances the performance of DCSwinLSTM in the task of spatiotemporal prediction of meteorological drought.

### Boundary-focused evaluation confirms improved drought-edge prediction

**Figure 8:**
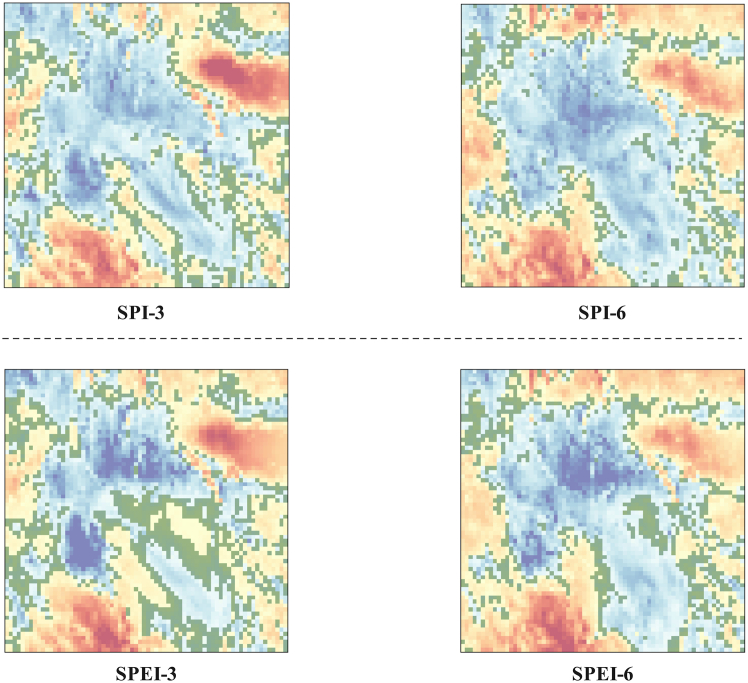
Illustration of the drought-boundary transition band used for boundary-focused evaluation For each task (SPI-3, SPI-6, SPEI-3, and SPEI-6), pixels within a low-index band at the dry end of the normalized field are highlighted (green), forming an edge-adjacent transition zone of block-style drought patches.

To provide an objective assessment of spatial boundary performance, we evaluate model errors specifically along drought-patch boundaries, where small spatial shifts can lead to noticeable boundary displacement. As illustrated in Figure 8, we identify a drought-boundary transition band on the normalized SPI/SPEI fields by selecting pixels at the dry end of the distribution, which highlight the edge-adjacent areas of block-style drought patches. We then compute MSE, MAE, and RMSE by comparing the predicted and ground-truth values only on these selected boundary pixels. The boundary-focused quantitative results for the SPI-3 task are summarized in Table 7. Among all compared methods, our models achieve the lowest boundary-region errors, with DCSwinLSTM obtaining the best MSE/MAE/RMSE on the selected boundary pixels and DCSwinLSTM-m performing very closely. This indicates that the proposed fusion improves boundary alignment, while the choice between single-gate and multi-gate updates mainly trades off efficiency and accuracy. Corresponding boundary-focused results for SPI-6, SPEI-3, and SPEI-6 are provided in Tables S1–S3.

**Table 7:** Boundary-focused quantitative comparison on SPI-3

Method	MSE↓	MAE↓	RMSE↓
ConvLSTM (NeurIPS’2015)	0.2618	0.4146	0.5117
PredRNN (NeurIPS’2017)	0.3805	0.4892	0.6169
MIM (CVPR’2019)	0.2773	0.4268	0.5266
PredRNN-V2 (TPAMI’2022)	0.5028	0.5636	0.7091
SimVP (CVPR’2022)	0.3118	0.4532	0.5584
SwinLSTM (ICCV’2023)	0.3702	0.4719	0.6084
TAU (CVPR’2023)	0.4362	0.5435	0.6605
WaST (AAAI’2024)	0.4445	0.5479	0.6667
**DCSwinLSTM (OURS)**	0.2286	0.3856	0.4782
**DCSwinLSTM-m (OURS)**	0.2366	0.3936	0.4864

## Discussion

In this study, we introduce an innovative feature fusion approach that integrates DConv and Swin transformer into the field of meteorological drought spatiotemporal series prediction. Extensive experiments are conducted on multi-indicator and multi-scale datasets. The results demonstrate that our model significantly outperforms existing methods in predicting drought data at different scales, benefiting from the effective fusion of boundary features and multi-scale spatial representations.

Evaluation on the ERA5 dataset shows that our two models achieve state-of-the-art accuracy and deliver the best or tied-best performance across all four prediction tasks (SPI-3, SPI-6, SPEI-3, and SPEI-6; Tables 1, 2, 3, and 4; Figures 1 and 2). The improvements are most evident on SPI, where DCSwinLSTM consistently reduces errors relative to both Transformer-based and memory-augmented baselines. On SPI-3, for instance, the MSE drops from 0.5119 to 0.4196 compared with SwinLSTM, accompanied by consistent reductions in MAE/RMSE and a PSNR gain of +0.86 dB, while also achieving a 46.2% MSE reduction over PredRNN. A similar pattern holds on SPI-6, where DCSwinLSTM reduces MSE from 0.3156 to 0.2775 versus SwinLSTM and by 53.4% versus PredRNN. On SPEI, DCSwinLSTM remains comparable to ConvLSTM on SPEI-3 and retains a small but consistent advantage on SPEI-6, while still outperforming PredRNN. We also note that the prediction errors on SPI-6 and SPEI-6 are generally lower than those on SPI-3 and SPEI-3. This is likely related to the stronger temporal smoothing and persistence of longer-timescale drought indices, which makes the corresponding monthly maps less variable and relatively easier to predict under the 1-month lead setting adopted in this study. To further examine generalization beyond drought-index forecasting, we additionally evaluate two models on the CIKM2017 precipitation benchmark, where they achieve competitive performance, with the lowest MSE and consistently strong CSI scores (Table 5), supporting that the proposed design is not limited to SPI/SPEI maps.

The observed performance differences are closely related to how each model balances temporal modeling with spatial representation. Drought-index fields commonly exhibit coherent patch-like structures with relatively sharp dry-wet transition zones, so small spatial shifts can manifest as noticeable boundary displacement. PredRNN-type models strengthen temporal memory, but their spatial modeling still relies primarily on convolutional receptive fields, which can lead to fragmented patches or shifted transitions when boundary geometry is irregular. Pure attention-based predictors capture longer-range spatial dependencies, yet they may smooth or weaken the continuity of block-style drought boundaries. By contrast, our design explicitly targets this boundary-sensitive regime: DConv adaptively samples along irregular patch edges while the Swin transformer provides multi-scale spatial context, and the recurrent update maintains temporal consistency over the input sequence.

To ground the qualitative comparison geographically, we analyze the enlarged domain in Figures 1 and 2, spanning the tropical-subtropical Indo-Pacific sector with strong land-sea contrasts and organized moisture-transport variability. In this region, drought often appears as coherent patches with sharp dry-wet transition zones, so boundary displacement corresponds to physically meaningful shifts of the transition band. The zoomed comparisons show that several baselines fragment or misplace patch boundaries, whereas DCSwinLSTM better preserves boundary integrity.

Targeted ablation experiments provide a mechanistic explanation for performance improvement. On SPI-3, replacing SwinLSTM with DCSwinLSTM reduces MAE/RMSE by 9.19%/8.67%, raises R^2^ by 16.2%, and improves PSNR by +0.79 dB (Table 6). Compared with ConvLSTM, DCSwinLSTM further achieves a 1.8% reduction in MSE and notable gains in R^2^ and PSNR, suggesting that stronger multi-scale spatial modeling is beneficial beyond convolution-recurrent baselines. Meanwhile DConvLSTM and ConvLSTM show very close performance in Table 6, so the benefit of introducing DConv alone appears incremental in this experiment. The clearer and more consistent gains are observed when DConv is integrated with the Swin transformer via lightweight Add fusion, suggesting that boundary-aware cues and multi-scale spatial modeling are complementary under small-sample training. Table 6 also shows that DCSwinLSTM-m can further improve the metrics under the same setting, consistent with the increased capacity of the standard multi-gate recurrent update.

Visualization results (Figure 7) indicate that DConv produces more spatially continuous feature responses around drought-patch boundaries than standard convolution, which helps preserve the integrity of block-style drought structures and reduces boundary fragmentation. After fusion with the Swin transformer features, the representation retains both boundary detail and broader multi-scale spatial context, supporting more coherent spatiotemporal evolution modeling. To complement this qualitative evidence with an objective boundary assessment, we further quantify prediction errors specifically along drought-patch boundaries using a drought-boundary transition band (Figure 8), and compute MSE, MAE, and RMSE restricted to these boundary pixels. The boundary-focused results for SPI-3 are summarized in Table 7, where our models achieve the lowest boundary-region errors among all compared methods, with DCSwinLSTM performing best on SPI-3 in this boundary-focused setting and DCSwinLSTM-m performing very closely.

In summary, we present DCSwinLSTM, a boundary-aware, feature-fusion framework for spatiotemporal prediction of global meteorological drought. Across four tasks (SPI-3, SPI-6, SPEI-3, and SPEI-6) derived from ERA5, DCSwinLSTM and its multi-gate variant consistently achieve the best overall performance among the selected baselines in terms of MSE, MAE, RMSE, R^2^, and PSNR. These gains indicate that combining boundary-sensitive feature extraction with hierarchical multi-scale spatial representation can better capture heterogeneous drought evolution, especially under limited-sample settings. The proposed framework therefore provides a practical solution for drought early warning and seasonal water-resource planning, and it can be extended in future work to broader time scales and additional hydroclimate variables.

### Limitations of the study

This study has several limitations. First, resampling to (256, 256) may distort geometry on a lon-lat grid, particularly at high latitudes. Second, rare extreme drought transitions remain difficult to learn under long-tailed, imbalanced data and index-based summaries of complex drivers. Third, stacked Swin blocks increase complexity relative to lightweight recurrent baselines. Future work will investigate more physically consistent grids or latitude-aware weighting and severity-aware training to improve extreme-event robustness while keeping the model efficient.

## Resource availability

### Lead contact

Requests for further information and resources should be directed to and will be fulfilled by the lead contact, Hui Liu (jacklh78@163.com).

### Materials availability

This study did not generate new unique reagents.

### Data and code availability

• This study analyzes existing, publicly available data, accessible at Figshare: https://doi.org/10.6084/m9.figshare.24485389.
• All original code generated for this study is available at GitHub: https://github.com/Htomwu/OpenSTL.
• Any additional information required to reanalyze the data reported in this study is available from the lead contact upon request.

## Acknowledgments

This work was supported by the 10.13039/100014472Scientific Research Foundation of Hunan Provincial Education Department (24C0104 and 24C0128; recipient, H.L.) and the 10.13039/501100013254National College Students Innovation and Entrepreneurship Training Program (202510538029; recipient, H.P.). We thank all members of the research team for helpful discussions and support during this study.

## Author contributions

H.P. conceived the study, developed the main idea and methodological framework, and wrote the original draft of the manuscript. C.W. conducted the experiments and performed the computational analyses. Y.D. and H.L. contributed to refining the methodology and interpretation, provided additional insights, and revised and edited the manuscript. All authors approved the final version of the manuscript.

## Declaration of interests

The authors declare no competing interests.

## STAR★Methods

### Key resources table

**Table:** 

REAGENT or RESOURCE	SOURCE	IDENTIFIER
**Deposited data**		

Processed global SPI/SPEI gridded dataset (1959–2022; SPI-3/SPI-6/SPEI-3/SPEI-6)	Figshare	https://doi.org/10.6084/m9.figshare.24485389

**Software and algorithms**		

DCSwinLSTM/DCSwinLSTM-m code (training & evaluation)	This paper	To be publicly released upon publication
Baseline implementations (ConvLSTM, PredRNN, MIM, PredRNN-V2, SimVP, SwinLSTM, TAU, WaST)	This paper	Included in the code release upon publication

**Other**		

NVIDIA GPU (V100)	This paper	Not applicable

### Experimental model and study participant details

This study did not involve human participants, animals, cell lines, or other experimental model systems. All experiments were conducted on gridded, monthly meteorological drought-index maps (SPI and SPEI) derived from global reanalysis data and organized as spatiotemporal sequences for one-step-ahead forecasting.

### Method details

#### Study region and dataset

The study region is global and we use the ERA5 dataset provided by the European Center for Medium-Range Weather Forecasts (ECMWF). This dataset employs the latest reanalysis techniques and has been shown in previous studies to more accurately reflect precipitation levels compared to other products.^36^^,^^37^ In this study, we use a published monthly SPI/SPEI dataset derived from ERA5 (1959–2022) released on Figshare: https://doi.org/10.6084/m9.figshare.24485389. The deep learning model is trained and evaluated on monthly SPI/SPEI maps (SPI-3, SPI-6, SPEI-3, and SPEI-6). Data spanning nearly 64 years, from 1959 to 2022, are used, with a horizontal resolution of 0.5 ° × 0.5 °.

#### Drought indices and accumulation scales

To assess drought conditions, this study employs two drought indices: the Standardized Precipitation Index (SPI)^38^ and the Standardized Precipitation Evapotranspiration Index (SPEI),^39^ both of which can be calculated from the ERA5 dataset for drought evaluation and prediction.

The SPI is primarily calculated based on precipitation, evaluating the deviation of precipitation within a given time window by fitting the long-term precipitation data to a probability distribution. Its computation typically involves fitting a Gamma distribution, which is then transformed into a standard normal distribution, allowing SPI values to be compared across different regions and time scales. In contrast, the SPEI not only considers precipitation but also incorporates evapotranspiration, providing a more comprehensive description of the climate water balance. The calculation of SPEI is based on the hydrological balance equation (precipitation minus potential evapotranspiration), followed by standardization in a manner similar to SPI.

For an accumulation timescale *k* (months), let *P*_*k*_ denote accumulated precipitation over k months. SPI is obtained by fitting a probability distribution *F*_*k*_(·) to the long-term series of *P*_*k*_ (commonly a Gamma distribution; with a correction for zero precipitation when applicable), computing the cumulative probability *μ* = *F*_*k*_(*P*_*k*_), and transforming it to the standard normal variate:

(Equation 1) $${SPI}_{k}=\Phi^{-1}\left(F_{k}\left(P_{k}\right)\right)$$

where Φ^−1^ (·) is the inverse CDF of the standard normal distribution.

For SPEI, the climatic water balance at month t is defined as:

(Equation 2) $$D_{t}=P_{t}-{PET}_{t}$$

and then obtain the k-month accumulation D_k_. A distribution G_k_ (·) is fitted to the long-term series of D_k_ (commonly a log-logistic distribution in the original SPEI formulation), compute the cumulative probability v = G_k_ (D_k_), and standardize it analogously:

(Equation 3) $${SPEI}_{k}=\Phi^{-1}\left(G_{k}\left(D_{k}\right)\right)$$

Compared with SPI, SPEI is more suitable for studying drought trends under the background of climate warming, as it captures the impacts of both reduced precipitation and increased evapotranspiration on water availability.^40^ To simultaneously capture te short- and medium-term evolution of drought, this study selects two time scales, 3 months and 6 months, and derives four drought indices: SPI-3, SPI-6, SPEI-3, and SPEI-6, which together provide a comprehensive representation of drought occurrence, development, and regional variation.We do not include SPI-1 or SPEI-1 in this study because our goal is to evaluate the model’s ability to forecast coherent regional drought patterns and boundary evolution in monthly maps. Compared with 3- and 6-month accumulation scales, 1-month indices typically exhibit stronger month-to-month fluctuations and more localized spatial intermittency, which can make regional pattern evaluation and architecture-level comparisons less stable. Therefore, we focus on 3- and 6-month indices as they provide smoother and more spatially coherent drought signals for benchmarking.^41^

Tables 8 and 9 present the specific values used to assess drought conditions with the SPI and SPEI indices.^42^

**Table:** Categorization of the severity of wet and dry events based on the SPI values

Categorization	Index value
Extremely wet	≥2.00
Very wet	1.50 to 1.99
Moderately wet	1.00 to 1.49
Near normal	−0.99 to 0.99
Moderately dry	−1.49 to −1.00
Severely dry	−1.99 to −1.50
Extremely dry	≤−2.00

**Table:** Categorization of the severity of wet and dry events often used for SPEI value

Categorization	Index value
Extremely wet	≥2.33
Very wet	1.65 to 2.32
Moderately wet	1.28 to 1.64
Mildly wet	0.84 to 1.27
Near normal	−0.83 to 0.83
Mildly dry	−1.27 to −0.84
Moderately dry	−1.64 to −1.28
Severely dry	−2.32 to −1.65
Extremely dry	≤−2.33

#### Data preprocessing

During the data preprocessing stage, samples with missing values across all time steps were first filtered out by detecting time points where all values were NaN, thereby removing invalid time nodes and enhancing the structural characteristics of the time series while reducing the impact of missing data on model training. For isolated missing values in the spatial dimension, mean imputation was applied, where the missing entries were filled with the mean spatial value at the corresponding time step, thus mitigating the abnormal disturbances caused by data incompleteness. To further stabilize the model training process and reduce training time, spatial downsampling was performed, adjusting the original resolution from (360, 720) to (256, 256). This square resolution was chosen to ensure consistent input formatting across the baseline models used in our comparison, as several image-based backbones and forecasting baselines assume or are configured for square inputs. Finally, min-max normalization was applied to map the data into the range of [0, 1] using the minimum and maximum values computed from the training set only, and the same scaling was applied to the validation and test sets to avoid temporal information leakage. After normalization, pixels with lower SPI/SPEI values (indicating relatively drier conditions) tend to be closer to 0, while pixels with higher SPI/SPEI values (indicating relatively wetter conditions) tend to be closer to 1. This process eliminates numerical scale differences across different regions and drought indices, and the model outputs are de-normalized back to the original SPI/SPEI units for quantitative evaluation and visualization.

#### Model overview

The framework design of the model in this paper is illustrated in Figure 9. The preprocessed data are first patch embedded into the backbone model. Boundary features and hierarchical multi-scale spatial features of the input meteorological drought data for four consecutive periods are then extracted using DConv and Swin Transformer, respectively. The two types of features extracted from the gridded SPI or SPEI data are subsequently fused by element-wise addition. Finally, the fused features are fed into an LSTM for training to obtain predictions of meteorological drought conditions for the next period.

**Figure:**
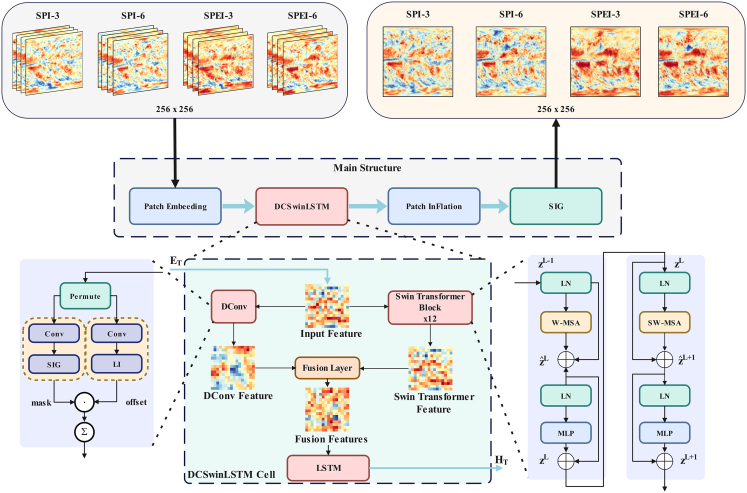
The integrated model is referred to as DCSwinLSTM, as its key components are DConv and SwinLSTM The DCSwinLSTM Cell serves as the main module of the model, where DConv is used to capture boundary features and the Swin transformer is responsible for extracting hierarchical multi-scale spatial features. These two types of features are fused and then fed into the LSTM for training and prediction.

We selected the SwinLSTM model and integrated it with the DConv module. The unique boundary feature extraction capability of DConv effectively addresses the limitation of previous drought prediction studies that overlooked the local similarity features of certain regions. By fusing the hierarchical multi-scale spatial features extracted by the Swin Transformer with the boundary features extracted by DConv, the model can better represent the data and further enhance prediction performance.

In the Figure 9, Patch Embedding converts each input drought-index map into a sequence of patch-level tokens for Transformer-based spatial modeling, while Patch Inflation restores the predicted token features back to a full-resolution map. SIG denotes the Sigmoid activation function used in the output stage to map the predicted features to the final drought-index output. For DConv, offsets and masks indicate the learned sampling displacements and modulation weights that guide the kernel to focus on irregular drought patch boundaries. In the Swin Transformer block, LN denotes Layer Normalization, W-MSA and SW-MSA denote shifted window-based multi-head self-attention, and MLP denotes the feedforward network. Here, E^T^ denotes the input feature tensor, z^L−1^,z^L^, z^L+1^ denote the input, output, and subsequent-layer feature representations of the l-th Swin Transformer block, respectively, and H^T^ represents the state feature processed by the LSTM.

DCSwinLSTM is an improved model based on the concept of ConvLSTM. ConvLSTM introduces convolutional operations to replace the fully connected operations between input-to-state and state-to-state transitions in traditional LSTM models, thereby effectively addressing the limitation of conventional models in capturing local spatiotemporal features. The core computational process of ConvLSTM can be described as follows:

(Equation 4) $$\left\{ \begin{array}{c} i_{t}=\sigma \left(W_{xi}*X_{t}+W_{hi}*H_{t-1}+b_{i}\right)\\ f_{t}=\sigma \left(W_{xf}*X_{t}+W_{hf}*H_{t-1}+b_{f}\right)\;\\ \;o_{t}=\sigma \left(W_{xo}*X_{t}+W_{ho}*H_{t-1}+b_{o}\right)\;\\ C_{t}=f_{t}\;\circ \;C_{t-1}+i_{t}\;\circ \;\tan\;h\left(W_{xc}*X_{t}+W_{hc}*H_{t-1}+b_{c}\right)\\ H_{t}=o_{t}\;\circ \;\tan\;h\left(C_{t}\right) \end{array}\right.$$

In the above equations, σdenotes the sigmoid activation function, ∗ represents the convolution operation, and ∘denotes the Hadamard element-wise product. i_t_, f_t_, o_t_ represent the input gate, forget gate, and output gate, respectively, while b denotes the bias for each gate. X_t_ denotes the data at time step t,*C*_*t*_ denotes the cell state at time t, H_t_ denotes the hidden state at time t. Compared with the fully connected structure in traditional LSTM, convolutional operations are more effective at extracting local spatial information. However, the model is limited in capturing long-range spatial dependencies. Therefore, in this study, DCSwinLSTM is designed by integrating the Swin Transformer with DConv: the former computes the similarity between features at different positions via a self-attention mechanism, while the latter captures regional similarity features. By fusing enhanced global spatial information with regional boundary features, the model’s capability for feature extraction and representation is improved. In the gating design adopted in SwinLSTM, all weights and biases in Equation 4 are omitted, resulting in the new Equation 5 as follows:

(Equation 5) $$\left\{ \begin{array}{c} {\;F}_{t}=i_{t}=f_{t}=o_{t}=\sigma \left(X_{t}+H_{t-1}\right)\\ C_{t}=f_{t}\;\circ \;C_{t-1}+i_{t}\;\circ \;\tan\;h\left(X_{t}+H_{t-1}\right)\\ H_{t}=o_{t}\;\circ \;\tan\;h\left(C_{t}\right) \end{array}\right.$$

In the simplified Equation 5, i_t_ = f_t_ = o_t_, where the input, forget, and output gates are merged into a unified control gate, referred to as the filter gate F_t_, This single-gate formulation is introduced primarily for efficiency, as it reduces parameter count and computational overhead, but it may reduce the model’s capacity to capture fine-grained temporal dynamics. To examine the impact of this simplification, we additionally implement a standard multi-gate LSTM (as Equation 4) variant (DCSwinLSTM-m) under the same setting and report the comparison in our experiments.

The overall structure of the DCSwinLSTM Cell is illustrated in Figure 10, while Figure 11 presents its multi-gate extension (DCSwinLSTM-m). Its innovative feature fusion approach enables further exploration of underlying patterns in the data across both spatial and temporal dimensions. By integrating the Swin Transformer with the DConv module, the Swin Transformer Block With Fusion (STBWF) module depicted in the figure is constructed. Specifically, taking the DCSwinLSTM cell as an example, at each time step we first concatenate (“Ⓒ”) the current input slice with the previous hidden state along the channel dimension. The concatenated tensor is passed to the linear projection (LP) and then to the STBWF module. Within STBWF, features from the Swin-Transformer and DConv branches are combined to produce a fused feature on the same spatial grid. This fused feature is then duplicated into two paths: one passes through tanh to form a candidate update for the cell state, and the other passes through sigmoid to produce a single gate. Through addition (“+”) operation, the candidate is added to the previous cell state, and through Hadamard Product operation (“⊙”), the result is modulated element-wise by the gate to obtain the updated cell state. The updated cell state is then passed through tanh, and the same gate (via duplication) is applied again at another Hadamard Product operation (“⊙”) to produce the new hidden state. Finally, the updated C_t_ and H_t_ are propagated to the next time step.

**Figure:**
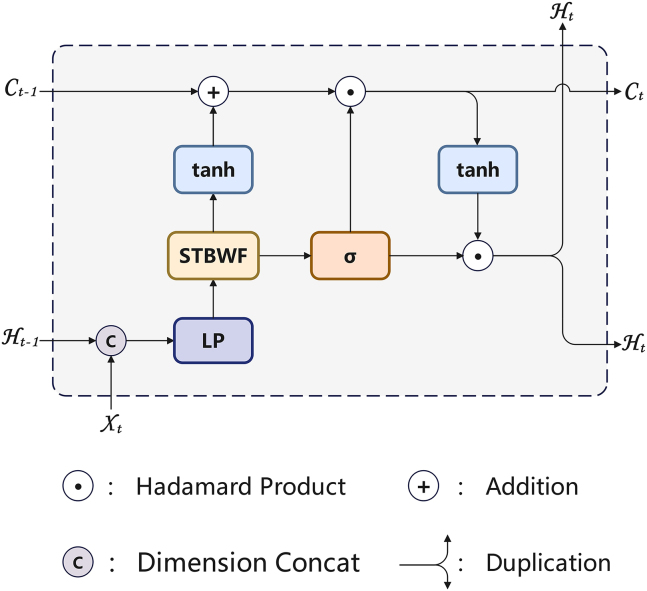
DCSwinLSTM cell

**Figure:**
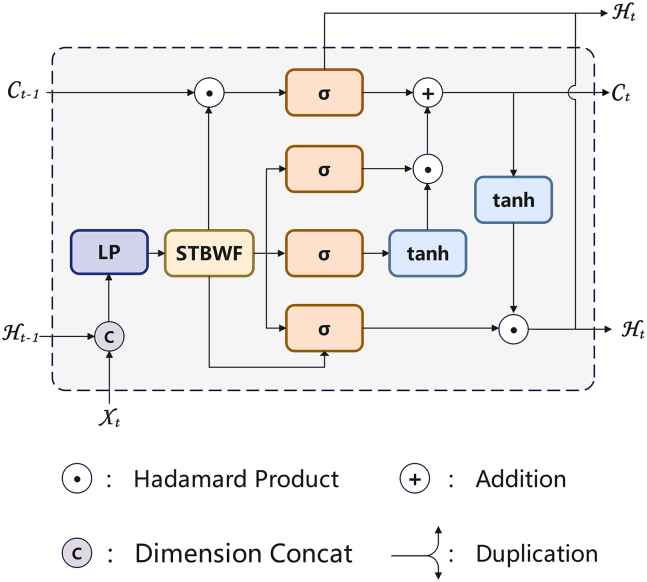
DCSwinLSTM-m cell

The overall workflow of DCSwinLSTM is as follows: the model first applies LP to reduce the dimensionality of the input features. Next, the features extracted by the Swin Transformer and DConv are interactively fused. Finally, a gating mechanism is employed to capture both long-term and short-term temporal dependencies, updating the cell state C_t_ and hidden state H_t_. The single-gate update used in DCSwinLSTM is given in Equation 6, while the DCSwinLSTM-m is summarized in Equation 7.

(Equation 6) $$\left\{ \begin{array}{c} F_{t}=\sigma \;\left(STBWF\left(LP\left(X_{t};H_{t-1}\right)\right)\right)\\ C_{t}=F_{t}\;\circ \;\left(\tan\;h\;\left(STBWF\left(LP\left(X_{t};H_{t-1}\right)\right)\right)+C_{t-1}\right)\\ H_{t}=F_{t}\;\circ \;\tan\;h\left(C_{t}\right) \end{array}\right.$$

(Equation 7) $$\left\{ \begin{array}{c} i_{t}=\sigma \;\left(\phi_{i}\left(STBWF\left(LP\left(X_{t};H_{t-1}\right)\right)\right)\right)\\ f_{t}=\sigma \;\left(\phi_{f}\left(STBWF\left(LP\left(X_{t};H_{t-1}\right)\right)\right)\right)\\ o_{t}=\sigma \;\left(\phi_{o}\left(STBWF\left(LP\left(X_{t};H_{t-1}\right)\right)\right)\right)\\ {\tilde{C}}_{t}=\left(\tan\;h\left(\phi_{c}\left(STBWF\left(LP\left(X_{t};H_{t-1}\right)\right)\right)\right)\right)\\ C_{t}=f_{t}\;\circ \;C_{t-1}+i_{t}\;\circ \;{\tilde{C}}_{t}\\ H_{t}=o_{t}\;\circ \;\tan\;h\left(C_{t}\right) \end{array}\right.$$

The other detailed formulations are presented in Equations 8 and 9.

(Equation 8) $$STBWF\left(X_{t};H_{t-1}\right)=Fusion\left(DConv\left(X_{t}\right),Swin\;Transformer\left(X_{t},H_{t-1}\right)\right)$$

(Equation 9) $$Fusion\left(Z_{Dconv}+Z_{Swin\;Transformer}\right)=Z_{Dconv}+Z_{Swin\;Transformer}$$

Where Z denotes the image features, ${\tilde{C}}_{t}$ denotes the candidate cell-state update. ϕ_i_, ϕ_f_, ϕ_o_, ϕ_c_ are learnable projections applied to Z to generate the corresponding gates and the candidate update.

#### Deformable convolution branch

Deformable Convolution^43^ is designed to address the limitations of conventional convolutional neural networks in handling geometric deformations and variations in object shapes. In our observations, meteorological drought grid maps often exhibit similar drought variation trends across different regions over time; capturing these patterns can further enhance the model’s feature extraction capability. Traditional convolution uses fixed-size kernels, which face obvious limitations when dealing with irregularly shaped or differently scaled targets. The core idea of Deformable Convolution is to adaptively adjust the sampling positions of the convolutional kernels based on the input features, enabling more flexible capture of important spatial features in images. Therefore, in this study, DConv is employed to extract boundaries from meteorological drought grid maps, identifying key regions with similar features. By dynamically adjusting the sampling locations of the convolution, greater weight is assigned to regions exhibiting similar drought trends, thereby allowing more precise and effective extraction of critical boundary features related to meteorological drought.

Since the extracted features in this study are applied within the Transformer framework, we employ one-dimensional DConv. The formulation of deformable convolution is shown in Equation 10.

(Equation 10) $$y\left(p_{0}\right)=\sum_{p_{n}\in R}w\left(p_{n}\right)\cdot x\left(p\right)$$

(Equation 11) $${p=p}_{0}+p_{n}+\Delta p_{n}$$

On this basis, by introducing the offset Δ*p*_*n*_, and utilizing one-dimensional linear interpolation as shown in Equation 12, the sampling positions are no longer restricted to regular intervals but are adaptively adjusted according to the input data. The offset Δ*p*_*n*_ enables the convolutional kernel to flexibly adjust its sampling locations based on data deformation, thereby capturing similarity features in drought-affected regions.

(Equation 12) $$x\left(p\right)=\sum_{q}g\left(q,p\right)\cdot x\left(q\right)$$

When the sampling position p is a non-integer value, one-dimensional linear interpolation is used to ensure that the information extracted from the input region is continuous. Here, *p* denotes the target position (Equation 11), qrepresents the neighboring integer positions, *x*(*q*) is the pixel value at position *q*, *g* (*a*,*b*) is the interpolation kernel function, as defined in Equation 13.

(Equation 13) g(a,b)=max(0,1−|a−b|)

*g* (*a*,*b*)is a smooth interpolation function used to compute the weights for interpolation at non-integer positions. It represents the similarity between position *p*and its neighboring integer positions *q*, thereby facilitating the identification of drought boundary regions.

#### Swin Transformer branch

In meteorological drought research, it is often necessary to process high-resolution remote sensing images that contain rich spatial information and multi-scale features. However, traditional Vision Transformers (ViT).^44^ which employ global self-attention, incur computational costs that grow quadratically with resolution, making them difficult to apply directly. To address this issue, the Swin Transformer,^26^ a variant inspired by ViT, was proposed. The Swin Transformer divides large images into local windows (W-MSA) and then uses shifted windows (SW-MSA) to enable information exchange across windows, thereby reducing computational complexity while efficiently capturing long-range spatial dependencies. Additionally, its hierarchical design enables multi-level abstraction and reconstruction of features at different resolutions. This approach is well suited for meteorological drought monitoring and prediction, as it facilitates the extraction of fine-grained, multi-scale spatial features in drought-affected regions, ultimately improving prediction accuracy. By combining multiple Swin Transformer Blocks with a simplified LSTM, the SwinLSTM model is constructed, which enhances the joint learning of long-range spatial information and temporal dependencies.

#### Training and inference workflow

Figure 12 illustrates the workflow of our proposed multi-scale meteorological drought prediction model, which consists of three stages: data processing, model training, and experimental validation.

**Figure:**
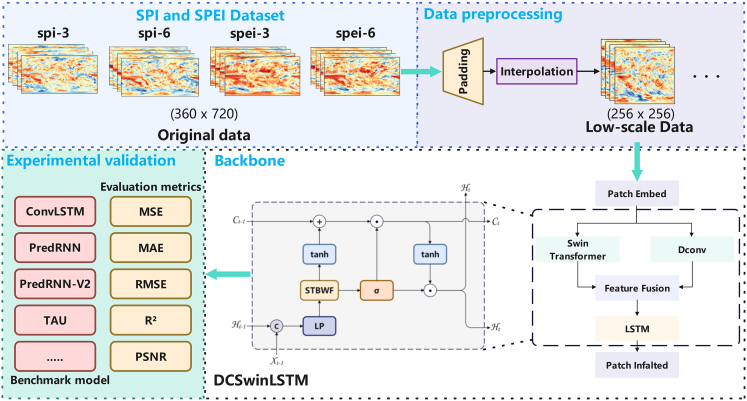
Workflow: After data preprocessing steps such as imputation and differencing to reduce data resolution, the processed data are simultaneously input into the Swin transformer and DConv for feature extraction The fused features are then passed to the LSTM to capture both short- and long-term dependencies for training and prediction. Finally, experimental validation and evaluation are conducted.

During the training phase, this study utilized a V100 GPU to train the models. Each model was trained for 200 epochs on different datasets corresponding to SPI-3, SPI-6, SPEI-3, and SPEI-6 at various time scales. For each experiment, four consecutive time-step data maps were used as input to predict the drought data map for the next time period. After training, the trained models were used for prediction, and the evaluation metrics were calculated accordingly.

#### Model implementation and training configuration

For each drought index and timescale, we formulate the task as one-step-ahead spatiotemporal map forecasting, where one step corresponds to a 1-month lead time. Given four consecutive monthly drought-index maps {*X*_*t*-3_,*X*_*t*-2_,*X*_*t*-1_,*X*_*t*_}, the model predicts the next map *X*_*t*+1_ at the same index and timescale. Each input map is first converted into patch-level tokens using a patch embedding layer with patch size 2 x 2, producing a token sequence that is processed by the proposed DCSwinLSTM cell. In the Swin Transformer branch, shifted-window self-attention is applied with window size 2 x 2, using an embedding dimension of 128, 12 Transformer blocks, and 4 attention heads. In parallel, the deformable convolution branch learns offsets and masks to adaptively focus on irregular drought patch boundaries. The two features are fused and propagated through the recurrent state to model temporal evolution over the input sequence, and the final prediction is reconstructed by patch inflation and mapped to the output drought-index field. The entire monthly sequence (1959–2022) is split chronologically into training, validation, and test subsets with a ratio of 6:2:2 to avoid temporal leakage.

Model training is performed with a batch size of 16 and an initial learning rate of 1 x 10-4. We adopt a OneCycle learning-rate scheduling strategy (OneCycleLR) to stabilize optimization and improve convergence. The model is trained on the training subset and monitored on the validation subset for model selection, and the final performance is reported on the held-out test subset. Moreover DCSwinLSTM and DCSwinLSTM-m parameter counts are 2.878M and 3.009M.

### Quantification and statistical analysis

The evaluation metrics used in this study include Mean Square Error (MSE), Mean Absolute Error (MAE), Root-Mean-Square Error (RMSE), R-squared (R^2^), and Peak Signal-to-Noise Ratio (PSNR). These metrics are used to comprehensively assess the differences between the predicted drought images and the ground truth images.

MSE represents the mean squared difference between the predicted and true values of all pixels in the image and is used to measure prediction accuracy. The calculation formula is shown in Equation 14.

(Equation 14) $$MSE=\frac{1}{n}\sum_{i=1}^{n}{\left(\hat{y_{i}}-y_{i}\right)}^{2}$$

Where $\hat{y_{i}}$ denotes the predicted value of the ith pixel in the image, and y_i_ denotes the true value of the i th pixel.

MAE represents the average absolute difference between the predicted and true pixel values in the image. The calculation formula is given in Equation 15.

(Equation 15) $$MA=\frac{1}{n}\sum_{i=1}^{n}\left|\hat{y_{i}}-y_{i}\right|$$

RMSE represents the root-mean-square of the differences between the predicted and true pixel values in the image. The calculation formula is shown in Equation 16.

(Equation 16) $$RMSE=\sqrt{\frac{1}{n}\sum_{i=1}^{n}{\left(\hat{y_{i}}-y_{i}\right)}^{2}}$$

R^2^ describes the goodness of fit of the model’s predictions to the image data. The calculation formula is provided in Equation 17.

(Equation 17) $$\left\{ \begin{array}{c} R^{2}=\frac{SSR}{TSS}\\ SSR=\sum_{i=1}^{n}{\left({\hat{y}}_{i}-{\bar{y}}_{i}\right)}^{2}\\ TSS=\sum_{i=1}^{n}{\left(y_{i}-{\bar{y}}_{i}\right)}^{2} \end{array}\right.$$

Here, Sum of Squares of the Regression (SSM) represents the sum of squared differences between the predicted pixel values and the mean pixel value of the original image, while Total Sum of Squares (TSS) represents the sum of squared differences between the original pixel values and their mean. denotes the mean value of all pixels in the ground truth image. ${\bar{y}}_{i}$ denotes the mean value of all pixels in the ground truth image.

PSNR is a widely used metric for evaluating image quality and measures the error between the predicted image and the ground truth image. The calculation formula is given in Equation 18.

(Equation 18) $$PSNR=10\times \mathrm{lg}\frac{{MaxValue}^{2}}{MSE}$$

Here, *MaxValue* denotes the maximum pixel value of the image.
