# Supplementary material for: DCSwinLSTM for spatiotemporal meteorological drought forecasting
Source: iScience. 2026 Apr 25;29(6):115902. doi: 10.1016/j.isci.2026.115902 (PMC13196577; doi:10.1016/j.isci.2026.115902)
Supplement: Document S1. Figures S1–S4 and Tables S1–S3 [file mmc1.pdf]

iScience, Volume *29*

Supplemental information

DCSwinLSTM for spatiotemporal meteorological drought forecasting

Haoxiang Peng, Chengrong Wu, Yuhao Du, and Hui Liu

Supplementary Figure 1. Qualitative comparison of SPI-6 predictions for the target date 2010-06-01 Rows show different models. Columns show (a) model prediction, (b) zoomed-in region (green box) for boundary comparison, and (c) signed error maps.

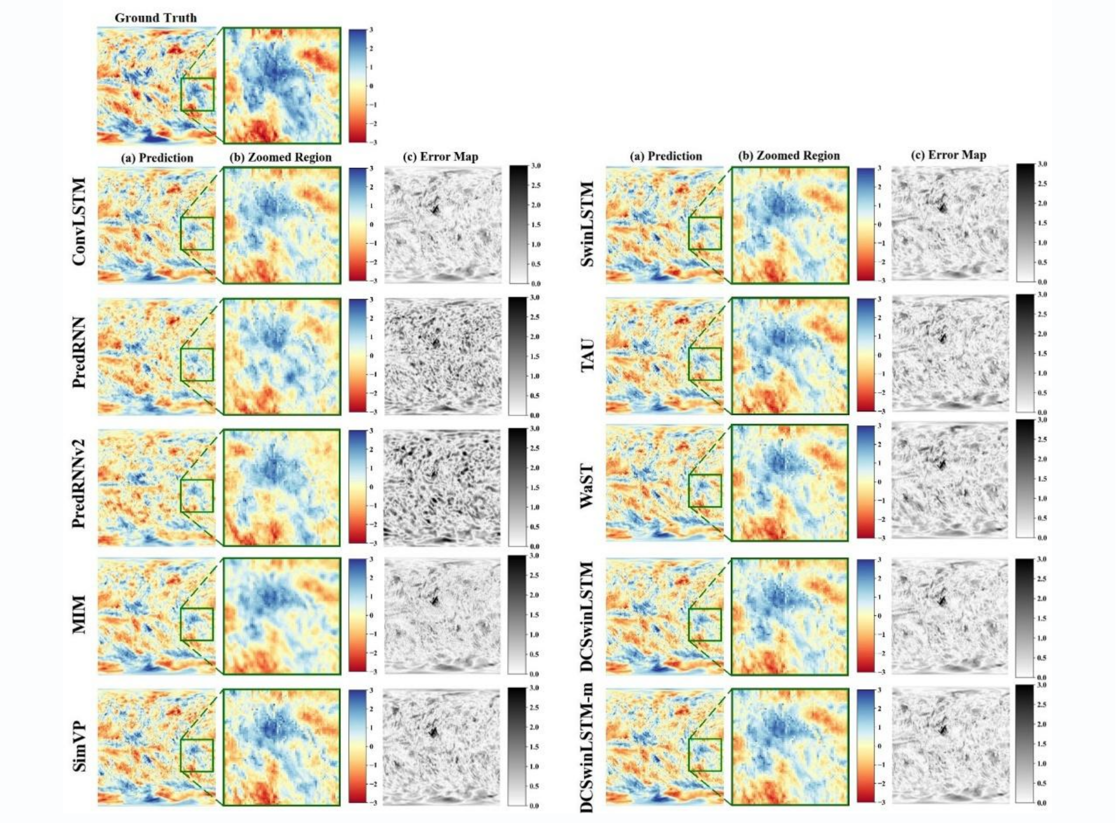

Supplementary Figure 2. Qualitative comparison of SPEI-6 predictions for the target date 2010-06-01 Rows show different models. Columns show (a) model prediction, (b) zoomed-in region (green box) for boundary comparison, and (c) signed error maps.

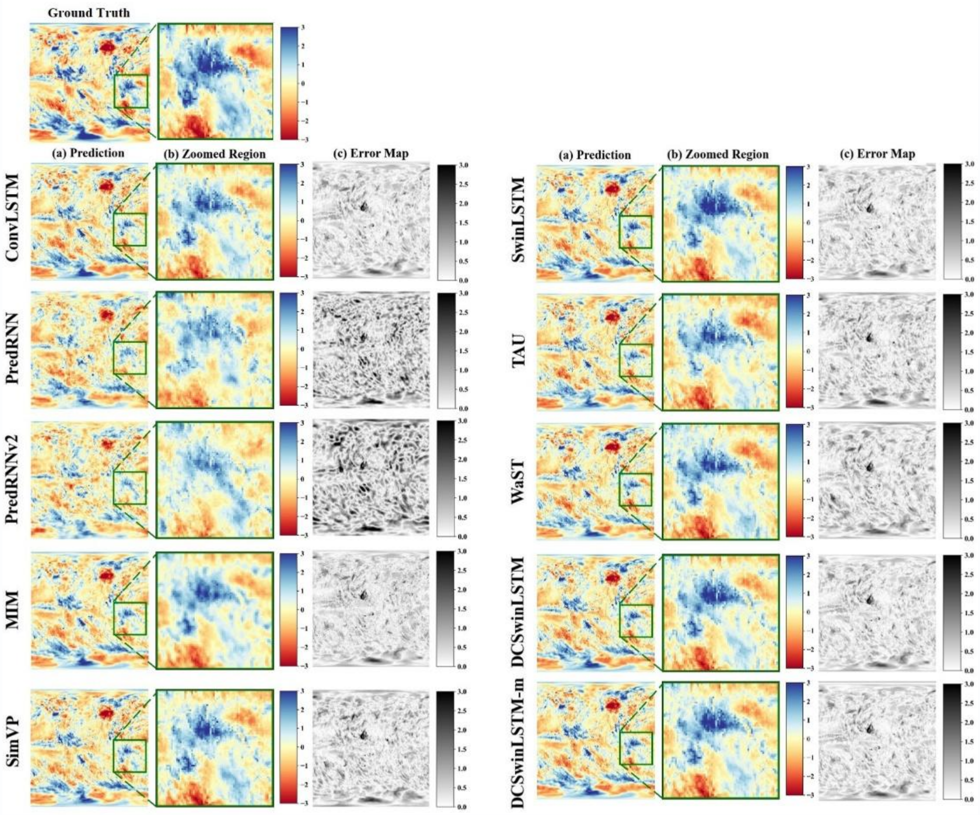

Supplementary Figure 3. Comparison chart of different model evaluation indicators. Using SPI-6 data from January 2010 to June 2022 for prediction and calculating MSE, RMSE, MAE, R^2, PSNR to draw line graphs.

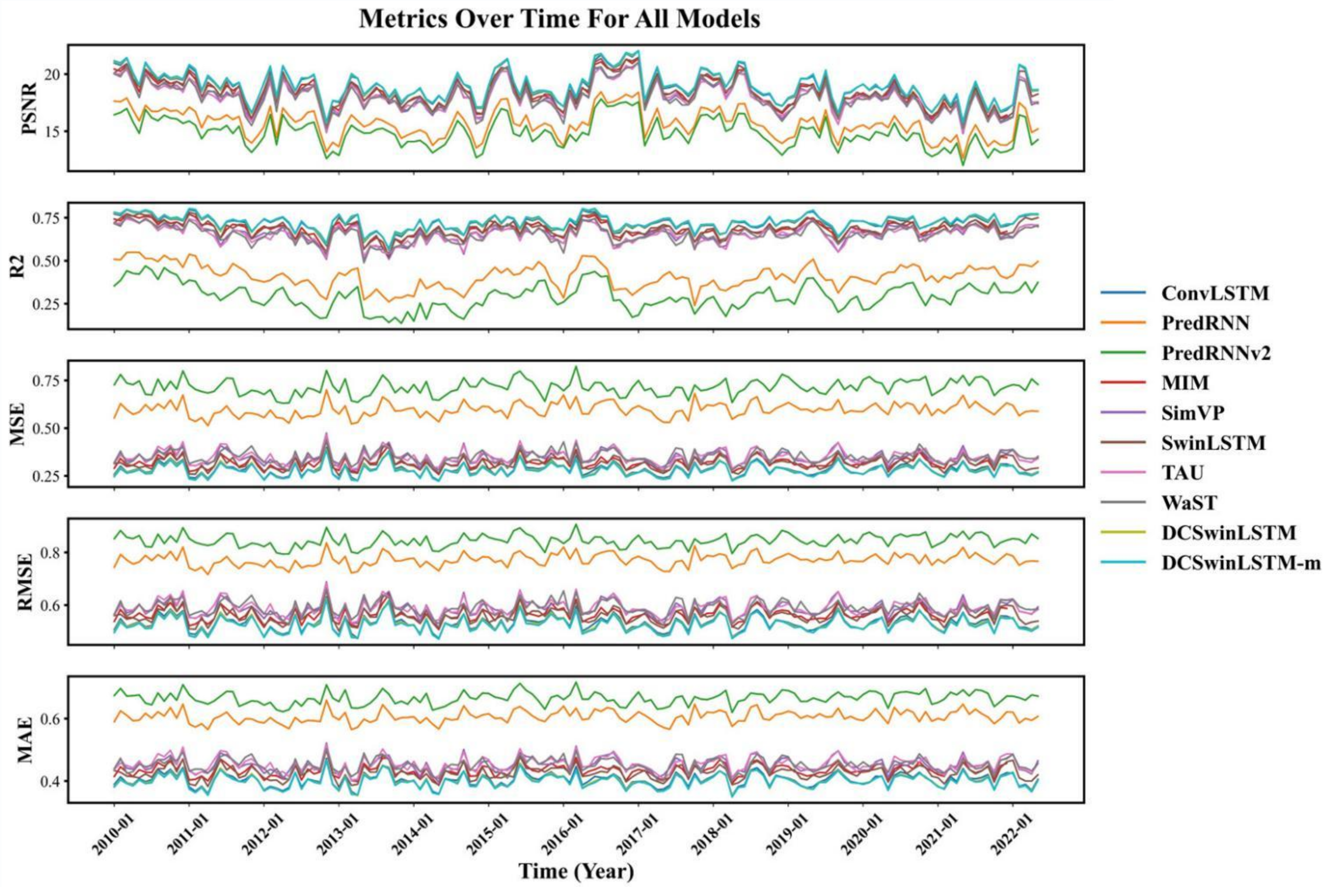

Supplementary Figure 4. Comparison chart of different model evaluation indicators. Using SPEI-6 data from January 2010 to June 2022 for prediction and calculating MSE, RMSE, MAE, R^2, PSNR to draw line graphs.

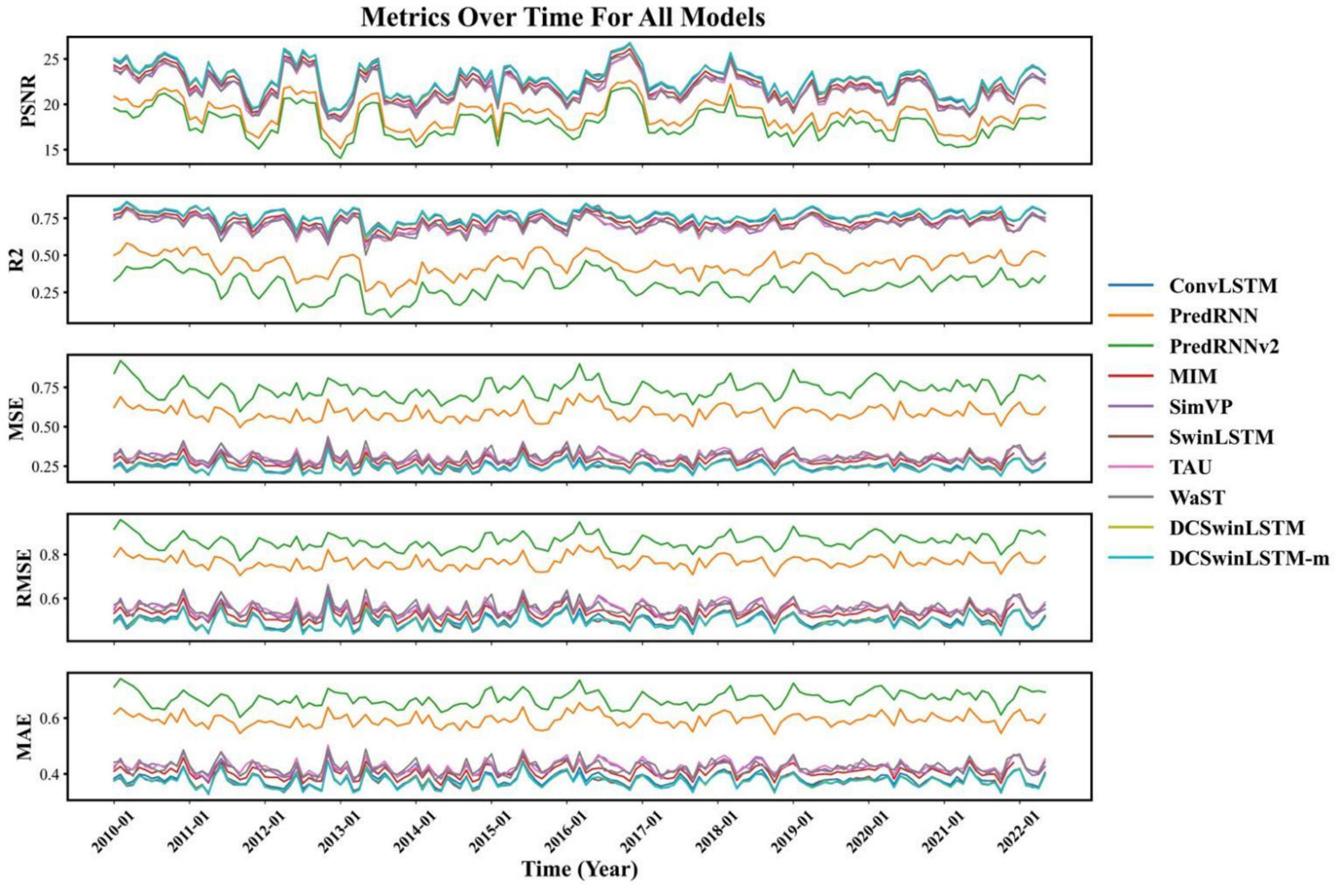

Supplementary Table 1. Boundary-focused quantitative comparison on SPI-6.

Method	MSE↓	MAE↓	RMSE↓
ConvLSTM(NeurIPS'2015)	0.1494	0.3068	0.3865
PredRNN(NeurIPS'2017)	0.3193	0.4509	0.5651
MIM(CVPR'2019)	0.1658	0.3270	0.4072
PredRNN-V2(TPAMI'2022)	0.5093	0.5802	0.7136
SimVP(CVPR'2022)	0.2042	0.3660	0.4519
SwinLSTM(ICCV'2023)	0.2056	0.3631	0.4535
TAU(CVPR'2023)	0.2172	0.3652	0.4661
WaST(AAAI'2024)	0.1703	0.3309	0.4127
**DCSwinLSTM(OURS)**	0.1483	0.3039	0.3851
**DCSwinLSTM-m(OURS)**	0.1409	0.2991	0.3753

Supplementary Table 2. Boundary-focused quantitative comparison on SPEI-3.

Method	MSE↓	MAE↓	RMSE↓
ConvLSTM(NeurIPS'2015)	0.1629	0.3216	0.4036
PredRNN(NeurIPS'2017)	0.2923	0.4247	0.5407
MIM(CVPR'2019)	0.3218	0.4638	0.5673
PredRNN-V2(TPAMI'2022)	0.4894	0.5604	0.6996
SimVP(CVPR'2022)	0.1753	0.3323	0.4187
SwinLSTM(ICCV'2023)	0.2169	0.3761	0.4657
TAU(CVPR'2023)	0.2229	0.3748	0.4722
WaST(AAAI'2024)	0.1224	0.2717	0.3499
**DCSwinLSTM(OURS)**	0.1280	0.2792	0.3578
**DCSwinLSTM-m(OURS)**	0.1384	0.2954	0.3720

Supplementary Table 3. Boundary-focused quantitative comparison on SPEI-6.

Method	MSE↓	MAE↓	RMSE↓
ConvLSTM(NeurIPS'2015)	0.1389	0.2876	0.3726
PredRNN(NeurIPS'2017)	0.3494	0.4776	0.5911
MIM(CVPR'2019)	0.1476	0.3034	0.3841
PredRNN-V2(TPAMI'2022)	0.3579	0.4726	0.5983
SimVP(CVPR'2022)	0.1674	0.3219	0.4091
SwinLSTM(ICCV'2023)	0.1291	0.2808	0.3592
TAU(CVPR'2023)	0.1633	0.3228	0.4041
WaST(AAAI'2024)	0.2016	0.3611	0.4491
**DCSwinLSTM(OURS)**	0.1358	0.2877	0.3686
**DCSwinLSTM-m(OURS)**	0.1249	0.2752	0.3534
